# Ab initio calculations of conduction band effective mass parameters of thermoelectric ${\hbox {Mg}}_{2} {\hbox {X}}_{1{-}x} {\hbox {Y}}_x$ (X, Y = Si, Ge, Sn) alloys

**DOI:** 10.1038/s41598-020-73277-9

**Published:** 2020-10-01

**Authors:** Juan M. Guerra, Carsten Mahr, Marcel Giar, Michael Czerner, Christian Heiliger

**Affiliations:** Institut für Theoretische Physik, Justus-Liebig-Universität (University of Giessen), 35392 Giessen, Germany

**Keywords:** Electronic properties and materials, Thermoelectrics

## Abstract

Since there are still research interests in the physical properties of quasi-binary thermoelectric ${\hbox {Mg}}_{2} {\hbox {X}}_{1-x}{\hbox {Y}}_{x}$ alloys, with X, Y = Si, Ge, Sn, we present an ab initio analysis that yields the relative formation energy and effective masses of the conduction bands, in the whole compositional range *x*. We base our calculations on the full-relativistic Korringa, Kohn and Rostocker (KKR) Green’s functions formalism within the coherent potential approximation (CPA). Formation energies, measured relative to the end ${\hbox {Mg}}_{2} \hbox {X}$ compounds, show no excess energy for the ${\hbox {Mg}}_{2} \hbox {Si} {-} {\hbox {Mg}}_{2} \hbox {Ge}$ substitution thus indicating a complete solubility. In contrast, concave and asymmetric formation energies for intermediate compositions in the ${\hbox {Mg}}_{2} \hbox {X} {-} {\hbox {Mg}}_{2} \hbox {Sn}$ alloys manifest a miscibility gap. With this basis, we compute and discuss the crossing of the conduction bands observed in *n*-type ${\hbox {Mg}}_{2} {\hbox {X}}_{1-x} {\hbox {Sn}}_x$ materials. We present direction- and band-dependent effective masses using a generalized single parabolic band effective mass approximation to discuss anisotropic effects, to interpret available experimental and theoretical data, and to predict intermediate and not yet published transport parameters on these alloys.

## Introduction

The physical and chemical properties of the *p*-type thermoelectric ${\hbox {Mg}}_{2} \hbox {X}$ (X = Si, Ge, Sn) semiconductors, and their intermediate alloys, are still under an intense research activity since they represent a rich scenario for green-technological applications^1–11^. Among the isoelectronic X elements, Si is considerably the cheapest, and despite of the scarceness and relatively higher costs of production, Ge and Sn are regarded as the best substitutions for alloying^12,13^. The thermoelectric performance of ${\hbox {Mg}}_{2} \hbox {Si}$ has shown to be substantially improved when Si is replaced by Sn due to an enrichment of its electronic density of states (DOS)—thus its power factor (PF)—and other induced detrimental effects on its phonons contribution to thermal transport^14^.

A long standing theoretical and experimental record of research has been presented, mostly concentrated in ${\hbox {Mg}}_{2} \hbox {X}$ crystals and ${\hbox {Mg}}_2 {\hbox {Si}}_{1-x} {\hbox {Sn}}_x$ alloys, and their fundamental properties are not yet completely understood^15–20^. For instance, the volumetric mismatch between ${\hbox {Mg}}_{2} \hbox {Si}$ (or ${\hbox {Mg}}_{2} \hbox {Ge}$) and ${\hbox {Mg}}_{2} \hbox {Sn}$ has been shown to cause thermodynamical instability—the *miscibility gap*—and therefore to induce ${\hbox {Mg}}_{2} \hbox {X}$-rich phases, clusters, and inhomogeneities^21–23^. The complete solubility of ${\hbox {Mg}}_{2} {\hbox {Si}}_{1-x} {\hbox {Ge}}_x$ has been successfully demonstrated^15,24–27^. In the remaining ${\hbox {Mg}}_{2} {\hbox {X}}_{1-x} {\hbox {Sn}}_x$ systems, X = Si, Ge, the experiments on solid solutions have shown that solubility depends rather upon the preparation method and experimental conditions^23,28,29^. No complete and stable solubility has been demonstrated^1^.

On the other hand, the improvement of the electronic properties has been associated to a degeneracy of the conduction bands controlled by the composition *x* in ${\hbox {Mg}}_{2} {\hbox {Si}}_{1-x} {\hbox {Sn}}_x$ and ${\hbox {Mg}}_{2} {\hbox {Ge}}_{1-x} {\hbox {Sn}}_x$, not present in ${\hbox {Mg}}_2 {\hbox {Si}}_{1-x} {\hbox {Ge}}_x$^14^. All alloys have shown to improve their thermoelectric performance, but only ${\hbox {Mg}}_{2} {\hbox {Si}}_{1-x} {\hbox {Sn}}_x$ and ${\hbox {Mg}}_{2} {\hbox {Si}}_{1-x} {\hbox {Sn}}_x$ present a peak of performance at the composition where the conduction bands cross, i.e., at the *crossing point*^30^. There is no yet clear consensus about the exact composition *x* and temperature at which the crossing occur, and the effect of alloying on the electronic states and transport properties. Its importance lies in the fact that controlling and engineering the degeneracy of the conduction bands through alloying represents a promising venue to improve the electronic PF without doping^31^.

Furthermore, there are still challenges in these systems, e.g., the search of the best dopants to design an efficient and environmentally friendly *n*-type sample—for the construction of a thermoelectric generator—and at the same time to suppress bipolar contributions to transport, etc.^32–36^.

We use Green’s functions based density functional theory (DFT)—ab initio—calculations to approach a description of the electronic properties of ${\hbox {Mg}}_{2} {\hbox {X}}_{1-x} {\hbox {Y}}_x$ alloys. To better understand the stability of these alloys and to improve the electronic DOS of these materials, we compute the relative formation energy and electronic bands effective masses using the coherent potential approximation (CPA) that describes perfectly random and homogeneous bulk alloys. We compare our calculations to available experimental and theoretical data and they show a very good agreement. This allows us to interpret and predict intermediate data not yet reported, specially in the case of ${\hbox {Mg}}_{2} {\hbox {Ge}}_{1-x} {\hbox {Sn}}_x$.

## Results

### Total energy calculations

Minimization of the *ground state* DFT total energy, with respect to small variations of the unit cell volume, allows us to find the structure equilibrium. We have reported that, besides the usual and system dependent underestimations of the local density approximation (LDA) to the exchange and correlation potentials, the minimized lattice constant of ${\hbox {Mg}}_{2} {\hbox {X}}_{1-x} {\hbox {Y}}_x$ varies non-linearly with the alloy composition *x*, therefore deviating from the empirical Vegard’s law^37^. This non-linearity is even more remarkable when the mismatch between the atomic masses of the substituted X and Y elements increases, i.e., for X = Si, Ge and Y = Sn^38^. This non-linearity is also reflected in the fundamental energy gaps and effective masses of the valence bands.

Total energy calculations have also been powerful to predict mechanical properties such as the bulk modulus and its derivative through a Murnaghan–Birch equation of state^39^. DFT total energy is the main ingredient to define the compounds’ formation energy to study and predict their stability through the construction of phase diagrams^40^. Under the DFT physical conditions, at $T=0\, \hbox {K}$ and $P=0\, \hbox {Pa}$, where the entropy contributions are negligible, the formation energy of a compound or alloy—its enthalpy of formation—permits to find the stable phases in the chemical space of compositions (*x*) through the so called convex hull, defined by the stable phases of the compounds. In turn, DFT formation energies have been widely useful to predict stable and meta-stable structures at using high-throughput computing and machine learning techniques^41,42^. Nonetheless, in order to study stability at room or higher temperatures, extra contributions coming from the entropy of phonons are present and possible phase transition may occur^43^.

Therefore, we take the DFT total energy to construct the relative formation energy diagram of ${\hbox {Mg}}_{2} {\hbox {X}}_{1-x} {\hbox {Y}}_x$ by assuming stable phases of the end compounds, i.e., at $x=0$ and $x=1$. Hence, positive (negative) relative formation energies mean that at zero temperature the compound is less (more) likely to exist compared to ${\hbox {Mg}}_{2} \hbox {X}$^44^.

We consider the on-site substitution between the X-elements at the edge of the unit cell, since this substitution (${\hbox {X}}_{\mathrm{Y}}$) is energetical and thermodynamically more likely to be present compared to the substitution ${\hbox {Mg}}_{\mathrm{Y}}$^32,45–48^. We take the DFT total energy *E* and define the formation energy of ${\hbox {Mg}}_{2} \hbox {X}$, obtained through the reaction

$$\begin{aligned} 2\hbox {Mg} + \hbox {X} \rightarrow {\hbox {Mg}}_{2} \hbox {X}, \end{aligned}$$

as

$$\begin{aligned} E_{form}=E({\hbox {Mg}}_{2} \hbox {X})-2E(\hbox {Mg})- E(\hbox {X}). \end{aligned}$$

In order to study the thermodynamic stability, the atomic chemical potentials ($\mu$) must be considered under some specific constrains of the bulk elements^49,50^. For these particular alloys, we generalize the equation of the formation energy of ${\hbox {Mg}}_{2} {\hbox {X}}_{1-x} {\hbox {Y}}_x$, obtained through the reaction

1 $$\begin{aligned} 2 \hbox {Mg} + (1-x) \hbox {X} + x \hbox {Y} \rightarrow {\hbox {Mg}}_{2} {\hbox {X}}_{1-x} {\hbox {Y}}_x, \end{aligned}$$

as

2 $$\begin{aligned} E_{Form}=E({\hbox {Mg}}_{2} \hbox {X})-2\mu (\hbox {Mg})- (1-x)\mu (\hbox {X})-x\mu (\hbox {Y}). \end{aligned}$$

The chemical potentials of Eq. (2) in the Mg-rich region are given by

3 $$\begin{aligned} \begin{aligned} \mu (\hbox {Mg})&=E(\hbox {Mg}), \\ \mu (\hbox {X})&=E({\hbox {Mg}}_2\hbox {X})-2E(\hbox {Mg}), \\ \text {and }\mu (\hbox {Y})&=E({\hbox {Mg}}_2\hbox {Y})-2E(\hbox {Mg}). \end{aligned} \end{aligned}$$

**Figure 1:**
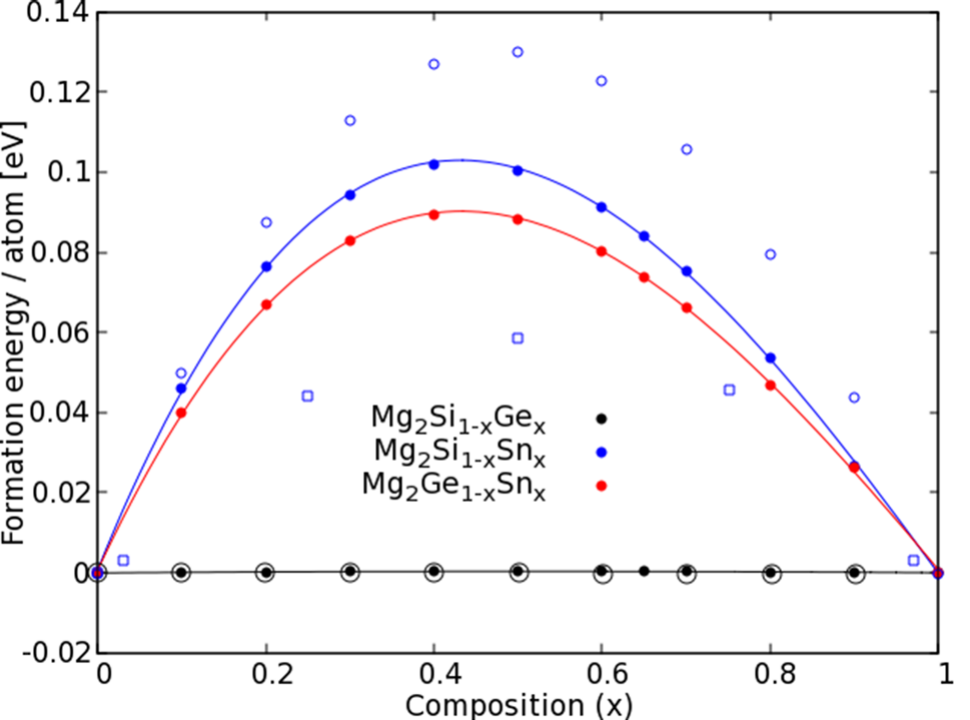
Relative formation energy per atom defined by Eq. (2), of the ${\hbox {Mg}}_{2} {\hbox {X}}_{1-x} {\hbox {Y}}_x$ alloys. Our calculations are presented using the solid dots as labeled, and the connecting lines correspond to a cubic polynomial fit. The empty circles^32^ and squares^24^ are calculations taken from literature, shown in black for ${\hbox {Mg}}_{2} {\hbox {Si}}_{1-x} {\hbox {Ge}}_x$ and in blue for ${\hbox {Mg}}_{2} {\hbox {Si}}_{1-x} {\hbox {Sn}}_x$.

Therefore, the relative formation energy given by Eqs. (2) and (3) vanishes at the end compounds, $x=0$ and $x=1$. We present our calculated relative formation energies for all quasi-binary ${\hbox {Mg}}_{2} {\hbox {X}}_{1-x} {\hbox {Y}}_x$ alloys in Fig. 1, in the compositional space *x*, using a resolution of $\Delta x=0.1$. We also compare our results with other theoretical findings previously reported. We note that the currently available data correspond to the ${\hbox {Mg}}_{2} {\hbox {Si}}_{1-x} {\hbox {Ge}}_{x}$ systems^32^, and ${\hbox {Mg}}_{2} {\hbox {Si}}_{1-x} {\hbox {Sn}}_x$^32,35^. No results have been reported so far about theoretical or experimental formation energies in ${\hbox {Mg}}_{2} {\hbox {Ge}}_{1-x} {\hbox {Sn}}_x$. We observe that the relative formation energy of the ${\hbox {Mg}}_2 {\hbox {Si}}_{1-x} {\hbox {Ge}}_x$ systems are nearly vanishing, and this result corroborates the observed solubility and thermodynamical stability at all compositions^15^. In contrast, in ${\hbox {Mg}}_{2} {\hbox {Si}}_{1-x} {\hbox {Sn}}_x$ and ${\hbox {Mg}}_{2} {\hbox {Ge}}_{1-x} {\hbox {Sn}}_x$, the positive formation energies indicate a lower stability and therefore a possible miscibility gap^1,21,24,51–54^. Our calculations reveal that the formation energies of ${\hbox {Mg}}_{2} {\hbox {Ge}}_{1-x} {\hbox {Sn}}_x$ lie below that of ${\hbox {Mg}}_{2} {\hbox {Si}}_{1-x} {\hbox {Sn}}_x$ and that both fit a cubic polynomial with maximal value at a content of $x\approx 0.43$ of Sn. We attribute this non-symmetric curve to the non-linear character of volumetric dependence of the alloys with composition^38^, in contrast to the symmetric behavior observed in other calculations presented in Fig. 1 that assumed that Vegard’s law holds. We also note that there is no direct experimental data to compare the formation energy since experiments are performed at room temperature, or higher, and it is also frequent to use temperature dependent phase diagrams^24^, or different approaches to investigate materials stability^54–56^. Solubility of these alloys is still under debate since, despite the existence of the miscibility gap, experiments have been reported for compositions between this gap^29^, and other experiments show instabilities based on variations of the measurements as the temperature increases and decreases^30^. Therefore we can interpret that the experimental conditions influence their stability and can lead to stable phases, as it is the case of e.g. the mechanical alloying technique^24,29,46,57^.

**Figure 2:**
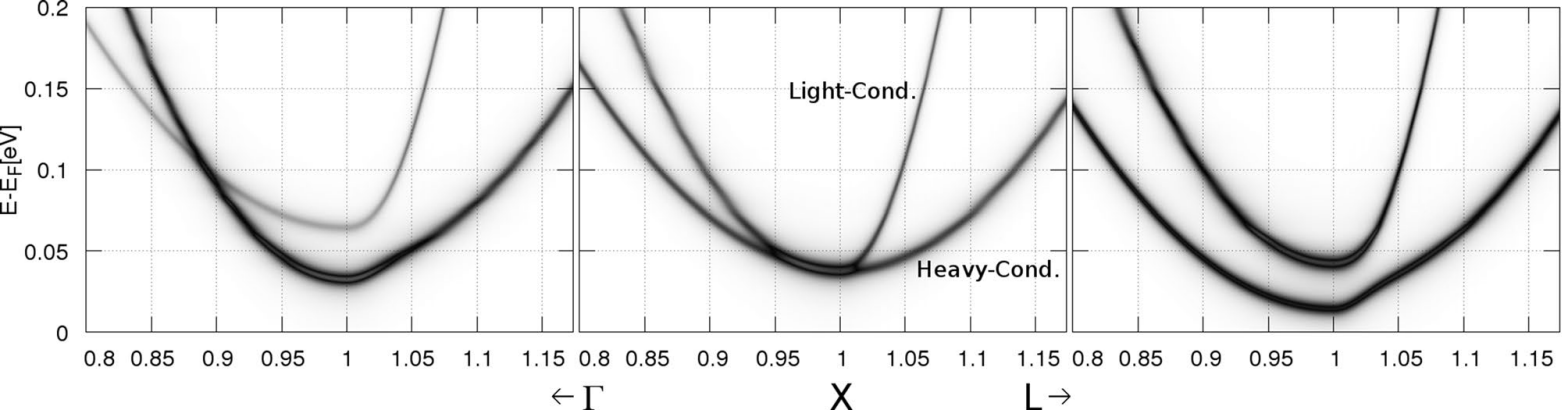
Low-lying light- and heavy-conduction bands of ${\hbox {Mg}}_{2} {\hbox {Ge}}_{1-x} {\hbox {Sn}}_x$ at compositions near the crossing, $x=0.65$ (left), $x=0.7$ (center), and $x=0.75$ (right), in the vicinity of the $\text {X}$-point [100] along $\text {X}-\Gamma$ and $\text {X}-L$, with *k* in units of $2\pi /a$ (dark gray corresponds to high BSF in a.u.).

### Effective mass parameters of the conduction bands

Within the CPA, using the total energy calculations presented in the Methods section and adequate structural parameters^38^, we compute the Bloch spectral function (BSF) to map the electronic bands and to extract information about the electronic states in the ${\hbox {Mg}}_{2} {\hbox {X}}_{1-x} {\hbox {Y}}_x$ alloys^58,59^. The CPA mimics the alloying effects on the electronic states a physical broadening, since it considers a uniform and randomly distributed bulk alloy. This broadening is even more pronounced for those bands that displace in energy as the composition changes, and thus fitting a rigid band with single *k*-value becomes a numerical challenge. We fit the bands using a Lorentzian profile and we test parabolicity to hold in an energy window up to $\approx \, 0.1\, \hbox {eV}$ ($k\approx 0.03 \, 2\pi /\hbox {a}$, a is the lattice constant^38^), as illustrated in Fig. 2^60^. Using these fitted *k*-values, we obtain the parabolic band effective mass at the $\text{ X }$ [100] point, along the $\vec k$-direction, using the formula

4 $$\begin{aligned} E(k)=E_0+\frac{\hbar ^2}{2m^*}(k- \text {X})^2, \end{aligned}$$

where $\hbar =h/2\pi$ is the reduced Plank’s constant, $m^*$ is the effective mass along $\vec k$ (*k* is the norm of $\vec k$), and $E_0$ corresponds to the indirect band gap. Hence, we extract the band effective mass ($m^*$) from the inverse of the curvature of the parabola. To measure anisotropic effects on transport, the experimental samples must be perfectly mono-crystalline and oriented along different crystallographic directions, as ruled by Eq. (4). Experiments are rather performed in randomly oriented samples and therefore the measurements correspond to an averaged effective mass over the reciprocal *k*-space. In order to avoid a very expensive integration, we compute the effective mass for each conduction band along different directions using Eq. (4). We have already successfully computed the effective masses of the conduction and valence bands located at the zone center—the $\Gamma$ point—of the BZ, using this generalized effective mass definition^38^. We have also found, that the (Lorentzian) broadening of the bands is on the order of $\sim \,1\%$ measured relative to the parabolic domain, and is more pronounced in the heavy conduction band as we present in Fig. 2.

In Fig. 2 we present the conduction bands of ${\hbox {Mg}}_{2} {\hbox {Ge}}_{1-x} {\hbox {Sn}}_x$ near the crossing point, that we determine to be at $x=0.7$. The bands of ${\hbox {Mg}}_{2} {\hbox {Si}}_{1-x} {\hbox {Sn}}_x$ present a similar behavior near the crossing point that we determine to take place at $x=0.65$. This crossing is therefore controlled by the composition *x* that also controls the size of the unit cell^38^. This implies, that at room temperatures the thermal expansion must be considered and the crossing points are expected to take place at a lower composition, i.e., near $x=0.6$ for ${\hbox {Mg}}_{2} {\hbox {Si}}_{1-x} {\hbox {Sn}}_x$^61–64^. We label the bands in Fig. 2 as *light*-conduction (LC) and *heavy*-conduction (HC) bands according to their effective mass. We note that both bands present a crossing along $\Gamma -\text {X}$ for compositions below $x=0.7$. Along $\text {X}-\text {L}$ we observe no crossing. This crossing moves down in energy by increasing the composition *x* of Sn, then the electron pockets merge at $x=0.7$^32^, and then, for larger compositions, the crossing disappears in both directions. We also note, that the curvature and the degeneracy of the bands are strongly dependent on the direction in the BZ. Along $\text {X}-\Gamma$, both bands show a wider degeneracy range, up to nearly $\sim \,0.05 (2\pi /a)$, compared to a range of nearly $\sim \,0.01 (2\pi /a)$ along $\text {X}-\text {L}$. This means, that the effective mass along $\text {X}-\Gamma$ must coincide for both conduction bands, while these bands are distinguishable along $\text {X}-\text {L}$ as their effective masses are. Additionally, the resulting masses must be different along both directions, especially for the LC band that is heavier along $\text {X}-\Gamma$.

**Figure 3:**
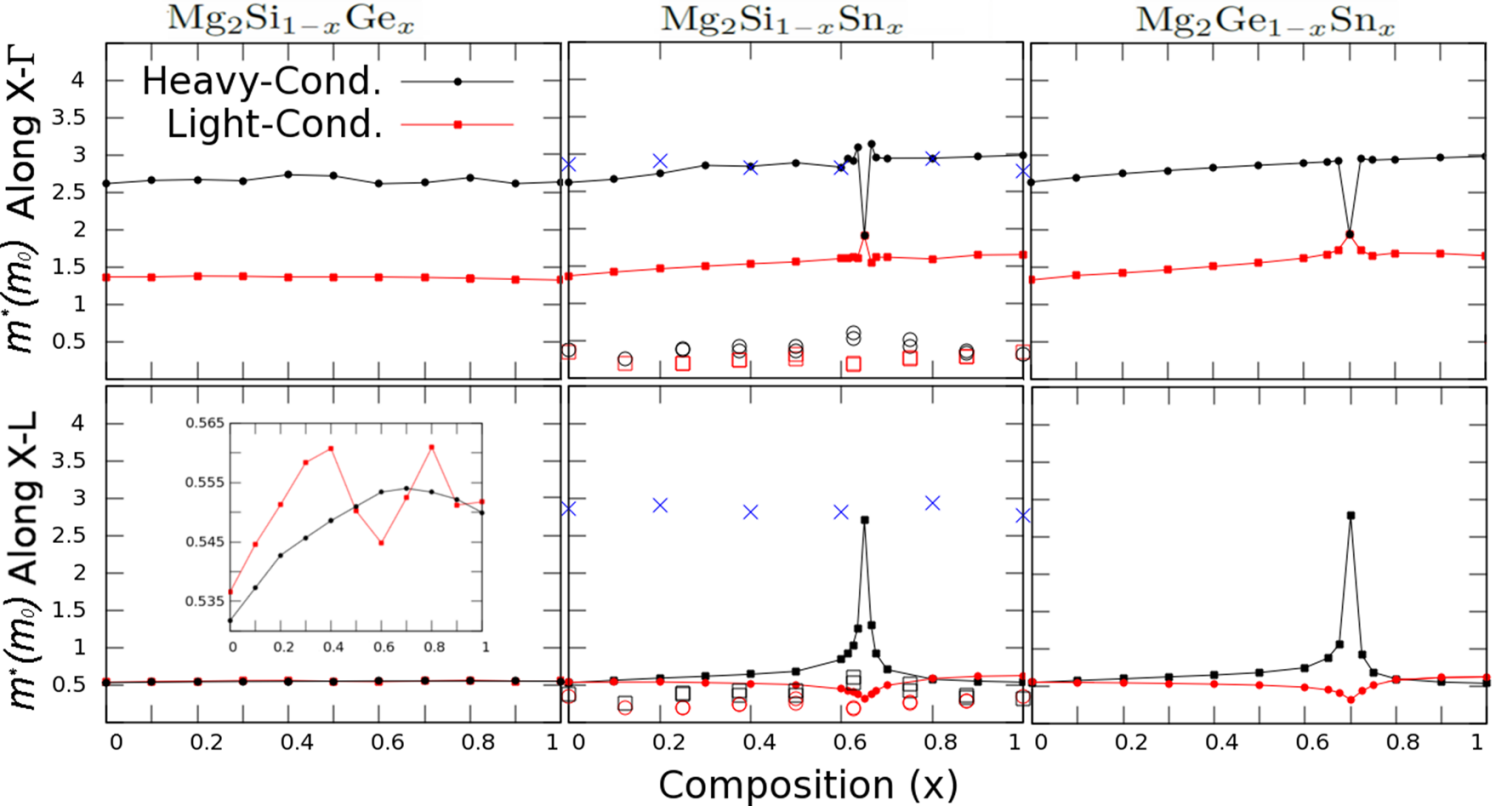
Parabolic band effective mass calculations corresponding to ${\hbox {Mg}}_{2} {\hbox {Si}}_{1-x} {\hbox {Ge}}_x$ (left), ${\hbox {Mg}}_{2} {\hbox {Si}}_{1-x} {\hbox {Sn}}_x$ (center), and ${\hbox {Mg}}_{2} {\hbox {Ge}}_{1-x} {\hbox {Sn}}_x$ (right), along the $X-\Gamma$ (top) and $X-L$ (bottom) directions, as function of the composition *x*. The available experimental data for ${\hbox {Mg}}_{2} {\hbox {Si}}_{1-x} {\hbox {Sn}}_x$ is presented using the blue X symbols^30^, while theoretical data is presented using the black and red empty symbols to represent the HC and LC effective masses, respectively^31,65^.

We present the computed effective masses ($m^*$), measured in units of the electron’s rest mass ($m_0$), as function of the composition for the ${\hbox {Mg}}_{2} {\hbox {X}}_{1-x} {\hbox {Y}}_x$ thermoelectric alloys in Fig. 3 along the directions given by Fig. 2. In the left column of Fig. 3 we show the ${\hbox {Mg}}_2 {\hbox {Si}}_{1-x} {\hbox {Ge}}_x$ system, in which the heavy- and light-conduction bands increase slow and constantly with the composition, and its value strongly depends on the direction. Along $\text {X}-\Gamma$, $m_{HC}^*$ and $m_{LC}^*$ keep a constant ratio of about 1.7, while along $X-L$ both bands are nearly similar, $m_{LC}^*\approx m_{HC}^*\approx 0.5m_0$. As shown in the inset of Fig. 3, and pointed out before, alloying effects are more remarkable in the HC band thus making it difficult to uniquely determine the effective mass.

In Fig. 3 we also present our computed effective masses for ${\hbox {Mg}}_{2} {\hbox {Si}}_{1-x} {\hbox {Sn}}_x$ (center) and ${\hbox {Mg}}_{2} {\hbox {Ge}}_{1-x} {\hbox {Sn}}_x$ (right) systems along $\Gamma -X$ (up) and $X-L$ (down). For ${\hbox {Mg}}_{2} {\hbox {Si}}_{1-x} {\hbox {Sn}}_x$, we include available experimental and theoretical data^30,31,65^. We point out, that the measured (DOS) effective masses correspond to ${\tilde{m}}^*=(N_v)^{2/3}m^*$, where $N_v$ is the degeneracy of the bands and $m^*$ corresponds to either a single pocket effective mass^31^, or an average of the bands^30^. The experimental data shown in Fig. 3 correspond to an average over the effective masses and does not manifest a degeneracy peak ($N_v=2$) at the crossing point. However, the experimental trend is very well described by our computed effective masses along $\text {X}-\Gamma$ considering that $N_v=1$, for *x* away from the crossing point. At this point, both bands are degenerate and have the same effective mass $m^*\approx 1.85m_0$, that multiplied by the degeneracy $N_v^{2/3}\approx 1.59$, coincides with the experimental value presented there.

Recently, using the DFT-supercell approach, and unfolding-back the BZ, band’s convergence in ${\hbox {Mg}}_{2} {\hbox {Si}}_{1-x} {\hbox {Sn}}_x$ was systematically studied as function of the unit cell volume—using Vegard’s law—to clarify the orbital composition of the bands and energy gaps^66^. On the other hand, the theoretical effective masses taken from literature and shown in Fig. 3 predict the crossing at compositions between $x=0.6{-}0.7$, nearly $x=0.625$, for ${\hbox {Mg}}_{2} {\hbox {Si}}_{1-x} {\hbox {Sn}}_x$, due to a limited resolution of the chemical-compositional space offered by the DFT-supercell approach^31,65^. These masses are not allowed to change for different orientations in the BZ and both data sources show similar numerical values. The trends of these masses as function of the composition are very well described by our calculations along $X-L$, especially the decrease reported in the LC mass by nearly a $40\%$, as we similarly report. Along $X-L$, the bands are distinguishable, and the computed masses from literature show an enhancement near to $45\%$ thus improving the absolute value of the PF by nearly 60–80$\%$. In these materials, the optimal thermoelectric performance is found at $T\approx 750\, \hbox {K}$, with an approximate dependency on the effective mass is given by $PF\sim (m^*)^{3/2}$. We concentrate at the crossing points and we include extra calculations to have a higher resolution close to this point. Our HC band is predicted to increase by a factor of $\sim \, 5$ and, without degeneracy, this value coincides with the experimentally measured mass. In ${\hbox {Mg}}_{2} {\hbox {Si}}_{1-x} {\hbox {Sn}}_x$, the Seebeck coefficient has been computed and enhances by nearly $50\%$^31^. Our computations represent the building block for macroscopic transport simulations using, e.g., the semi-classical multi-band approach to transport—the Boltzmann transport equation—within the relaxation time approximation. Transport simulations require extra approximations and, e.g., the consideration of the electronic mobility to account for bipolar effects^60,67^. We extrapolate this study to predict the transport parameters for ${\hbox {Mg}}_{2} {\hbox {Ge}}_{1-x} {\hbox {Sn}}_x$ that show a similar behavior compared to ${\hbox {Mg}}_{2} {\hbox {Si}}_{1-x} {\hbox {Sn}}_x$ and have not been yet published.

## Discussion

We have used the DFT-based Green’s functions KKR formalism in the full relativistic case, within the CPA for describing the electronic properties of the ${\hbox {Mg}}_{2} {\hbox {X}}_{1-x} {\hbox {Sn}}_x$ alloys. After carefully study of the structural details, within the LDA, we compute the formation energy that sheds lights on structural stability, specially about the existing miscibility gap between ${\hbox {Mg}}_{2} \hbox {X}$ (X=Si,Ge) and ${\hbox {Mg}}_{2} \hbox {Sn}$. We conclude that, in spite of the existence of a experimentally reported miscibility gap in the ${\hbox {Mg}}_{2} {\hbox {X}}_{1-x} {\hbox {Sn}}_x$ systems, many experiments have reported their stability during the measurements but many others have shown inconsistent results. Therefore, the solubility of these alloys depend on the experimental conditions that overcome the excess of formation energy. We predict the exact composition at which the conduction bands merge in ${\hbox {Mg}}_{2} {\hbox {X}}_{1-x} {\hbox {Sn}}_x$ and we interpret other experimental and theoretical results by fitting a detailed band structures near the crossing points. Our calculations have shown a very good agreement with other results and allow us to predict intermediate data not published so far.

## Methods

The computational details of the present work are based on a previous report^38^. We use the Green’s functions based Korringa–Kohn–Rostocker (KKR) formalism, in full-relativistic description^68^, and within the local density approximation (LDA) for treating the exchange-correlation potential^69^. For the electronic properties of a homogeneous alloy phase, we map the complex band structure using a Bloch spectral density function (BSF) defined within the coherent potential approximation (CPA)^58^. The CPA is a very efficient approach to the alloy problem since it strongly reduces computational costs without folding the BZ. Since CPA considers uniformly random bulk alloys to construct an efficient complex-potential, CPA describes the broadening on the bands due to chemical disorder. This task would be in principle very difficult to reach using a supercell-DFT approach since it would require the computation over a very large—in principle infinite—number of configurations. Moreover, we are allowed to define a randomly fine chemical space of compositions near the crossing points, which is also a very hard task for the supercell approach. We assume the anti-fluorite ($Fm{\bar{3}}m$) crystallization of the ${\hbox {Mg}}_{2} \hbox {X}$ compounds and their intermediate alloys, with lowest conduction bands at the *X*-point. The X = Si, Ge, Sn atoms form a diamond cubic structure while the Mg atoms have a hexagonal close-packed structure^1^. Our results have shown a very good agreement with available experimental data and represent a good source of—to the date—unavailable intermediate data in these alloy systems. Therefore, we retain the computational scheme to extract the relative formation energy from the total energy calculations, within the atomic sphere approximation (ASA).
